# Fabrication and Characterization of Quad-Component Bioinspired Hydrogels to Model Elevated Fibrin Levels in Central Nervous Tissue Scaffolds

**DOI:** 10.3390/gels10030203

**Published:** 2024-03-17

**Authors:** Ana M. Diaz-Lasprilla, Meagan McKee, Andrea C. Jimenez-Vergara, Swathisri Ravi, Devon Bellamy, Wendy Ortega, Cody O. Crosby, Jennifer Steele, Germán Plascencia-Villa, George Perry, Dany J. Munoz-Pinto

**Affiliations:** 1 Engineering Science Department, D. R. Semmes School of Science, Trinity University, San Antonio, TX 78212, USA; adiazlas@trinity.edu (A.M.D.-L.); mmckee@trinity.edu (M.M.); ajimene1@trinity.edu (A.C.J.-V.); wortega@trinity.edu (W.O.); 2 Biology Department, D. R. Semmes School of Science, Trinity University, San Antonio, TX 78212, USA; sravi@trinity.edu; 3 Chemistry Department, D. R. Semmes School of Science, Trinity University, San Antonio, TX 78212, USA; dbellamy@trinity.edu; 4 Department of Physics, Southwestern University, Georgetown, TX 78626, USA; crosbyc@southwestern.edu; 5 Physics and Astronomy Department, D. R. Semmes School of Science, Trinity University, San Antonio, TX 78212, USA; jsteele@trinity.edu; 6 Department of Neuroscience, Developmental and Regenerative Biology, College of Sciences, The University of Texas at San Antonio (UTSA), San Antonio, TX 78249, USA; german.plascenciavilla@utsa.edu (G.P.-V.); george.perry@utsa.edu (G.P.); 7 Neuroscience Program, D. R. Semmes School of Science, Trinity University, San Antonio, TX 78212, USA

**Keywords:** interpenetrating polymer networks, bioinspired scaffolds, fibrin hydrogels

## Abstract

Multicomponent interpenetrating polymer network (mIPN) hydrogels are promising tissue-engineering scaffolds that could closely resemble key characteristics of native tissues. The mechanical and biochemical properties of mIPNs can be finely controlled to mimic key features of target cellular microenvironments, regulating cell-matrix interactions. In this work, we fabricated hydrogels made of collagen type I (Col I), fibrin, hyaluronic acid (HA), and poly (ethylene glycol) diacrylate (PEGDA) using a network-by-network fabrication approach. With these mIPNs, we aimed to develop a biomaterial platform that supports the in vitro culture of human astrocytes and potentially serves to assess the effects of the abnormal deposition of fibrin in cortex tissue and simulate key aspects in the progression of neuroinflammation typically found in human pathologies such as Alzheimer’s disease (AD), Parkinson’s disease (PD), and tissue trauma. Our resulting hydrogels closely resembled the complex modulus of AD human brain cortex tissue (~7.35 kPa), promoting cell spreading while allowing for the modulation of fibrin and hyaluronic acid levels. The individual networks and their microarchitecture were evaluated using confocal laser scanning microscopy (CLSM) and scanning electron microscopy (SEM). Human astrocytes were encapsulated in mIPNs, and negligible cytotoxicity was observed 24 h after the cell encapsulation.

## 1. Introduction

Hydrogels are a valuable tool in tissue engineering because their biochemical and mechanical properties can be tailored to closely resemble key characteristics of the extracellular matrix (ECM) of various tissues [1,2,3]. The ECM is a dynamic and three-dimensional network of macromolecules that provides structural and functional support to cells and tissues. In the central nervous system (CNS), the ECM plays a pivotal role in various physiological processes, including normal tissue function, tissue regeneration, and the regulation of acute and chronic inflammation [4,5]. Pathological conditions such as tissue damage due to aging, traumatic brain injury (TBI), and neurodegenerative disorders like Alzheimer’s disease (AD) and Parkinson’s disease (PD) are often associated with ECM modifications involving remodeling and tissue degradation [6,7]. Therefore, understanding these dynamics is crucial for developing effective treatment strategies that can slow damage to nervous tissue and diminish the progression of CNS pathologies. 

ECM remodeling and degradation influence the release of cytokines and growth factors, which contribute to downstream inflammatory signaling and CNS tissue function and regeneration [8]. In response to immunomodulatory molecules released from stressed, injured, or dying cells, glial and endothelial cells adjacent to the injured tissue proliferate and activate [9]. The propagation of inflammatory signaling fosters further ECM remodeling, initiating a positive feedback loop that results in chronic inflammation. Astrocytes, specialized glial cells, play a critical role in CNS function by maintaining and regulating brain homeostasis, supporting neuronal health, regulating synaptic function, and responding to injuries or diseases [10,11]. Under normal physiological conditions, astrocytes exist in a quiescent state. However, changes in the ECM composition due to injury, inflammation, neurodegenerative diseases, or other pathologies can activate these astrocytes, initiating an immune response and aiding in the repair process. This activation transforms astrocytes from a quiescent to a reactive state in a process known as astrogliosis [12]. While astrogliosis can be beneficial in containing damage, excessive or prolonged activation may contribute to neuroinflammation and scar formation [13].

Several pathological changes in the ECM characterize the neuroinflammatory response. Hyaluronic acid (HA) degrades from high molecular weight (HMW) polymer into biologically active low molecular weight (LMW) fragments [14]. Additionally, pathological disruption of the blood-brain barrier (BBB) leads to abnormal fibrin deposition into the CNS [15,16]. Significant research has been conducted to explore the role of the ECM in neurological disorders. Despite these efforts, the impact of these pathological ECM changes on cell behavior is not well understood, so we seek to understand these in an in vitro study using hydrogels.

In recent studies, hydrogel-based ECM models have been developed by designing multicomponent interpenetrating polymer networks (mIPNs). These hydrogels consist of at least three natural and synthetic polymer networks combined to generate a single interlocked macromolecular structure to synergize the key characteristics of each component. In this way, physical and biochemical properties can be tuned to closely resemble a given tissue target. Fibrous proteins such as collagen and components such as hyaluronic acid have been previously employed in modeling in vitro systems of brain cortex tissue [17,18]. Furthermore, synthetic polymers such as polyethylene glycol (PEG) have been used to control the physical stability and the mechanical properties of the constructs. Moreover, PEG-based hydrogels are resistant to cell-mediated contraction and degradation, and their well-established biocompatibility and tunable biological behavior make them a very useful tissue-engineering platform [19,20]. For example, Saleh et al. developed mIPNs by combining varying ratios of collagen and thiolate HA crosslinked with poly (ethylene glycol) diacrylate (PEGDA) to investigate the synergistic effects of ECM composition and stiffness on astrocyte cell response [21]. The study revealed that soft hydrogels with lower HA concentrations increased cell spreading and astrocyte reactivity. Additionally, in previous studies, Van Drunen et al. and Jimenez-Vergara et al. also designed 3D platforms using mIPNs comprising of collagen, HA, and PEGDA to support the in vitro culture of CNS cells and have been demonstrated to be a valuable tool for studying the effects of HA degradation on the inflammatory response of human astrocytes [22,23]. These previous studies indicated that a reduction in the concentration of HA-HMW led to a decrease in the total length of astrocytic processes and a significant increase in the expression of reactive and inflammatory markers [23].

In this work, we increased the complexity of our current mIPN model by incorporating a fibrin network. Fibrin, a fibrous protein derived from fibrinogen via the enzymatic action of thrombin, plays a pivotal role in the inflammatory response of CNS tissue to neurovascular injury and neural degeneration [24]. Herein, we fabricated the mIPNs using a network-by-network fabrication approach, incorporating collagen type I, HA, fibrin, and PEGDA into a single engineered scaffold. By modulating PEGDA molecular weight (MW) and concentration, we fabricated a library of hydrogels and identified the formulations that closely matched the mechanical performance of AD human brain cortex tissue while mimicking changes in ECM composition that follow tissue damage. Furthermore, we characterized the microarchitecture of mIPNs using confocal laser scanning microscopy (CLSM) and scanning electron microscopy (SEM), successfully encapsulated human astrocytes into the scaffolds, and demonstrated that cell viability was at least 95% 24 h post-encapsulation. In addition, the proposed mIPNs promote the adoption of star-shaped morphology in human astrocytes in a 3D context. In summary, we have developed a cytocompatible in vitro 3D culture platform that allows for the control and adjustment of fibrin composition while closely matching the complex modulus of AD human brain cortex. This novel biomaterial could be used to further elucidate distinct stages of reactive and inflammatory phenotypes in human astrocytes, providing a valuable 3D platform for studying key aspects of the physiology and the progression of neurological pathologies.

## 2. Results and Discussion

### 2.1. Fabrication of mIPNs

While traditional IPNs and semi-IPNs are fabricated using two components, there has been an increase in the use of interpenetrating networks comprising more than two components in tissue engineering [25]. This is due to the need to create more biomimetic and functional environments that promote cellular growth and functionality. In previous studies, hydrogels have been developed using components such as collagen, HA, fibrin, and PEGDA, either individually or in combination with other materials [26,27,28]. For example, Li et al. fabricated a tri-component interpenetrating polymer network comprised of collagen, HA, and chondroitin sulfate (CS) as scaffolds for brain tissue engineering. The study revealed that Col-HA and Col-CS-HA scaffolds selectively enhance neurogenesis compared to a hydrogel consisting of only collagen and may be advantageous in tissue-engineering therapy for brain repair [17]. Similarly, Bindi et al. developed a bioinspired hydrogel mimicking skin’s extracellular matrix (ECM) based on collagen type I, hyaluronic acid, 4S-StarPEG, and fibrin. The hydrogel was optimized for porosity and rheological properties, showing improved viscoelasticity and promoting cell growth, presenting a promising biomimetic matrix for diverse soft tissue-engineering applications [29]. In previous studies, our research team has been able to successfully fabricate and characterize scaffolds containing Col I, HA, and PEGDA into a single hydrogel [22,23]. In this work, we have increased the complexity of the engineered structure by intruding a fourth component, further expanding the bioactivity of the mIPNs and the potential use of this platform as a 3D cell culture system. Herein, multicomponent interpenetrating networks were fabricated using a network-by-network approach. The fabrication process involved creating each network using orthogonal crosslinking mechanisms similar to the layer-by-layer approach used for complex surfaces. The sequential construction of individual networks, rather than forming the scaffolds from a polymer blend in a single polymerization step, facilitated dynamic and chronological control and regulation over the crosslinking process, the scaffold formation, and the resulting biophysical properties of the network. The use of this system facilitates control over the degree of cell spreading and the mechanical properties of multiple tissue targets.

The use of Col I as one of our proposed networks introduces a significant constraint since collagen fibrils form rapidly at a neutral pH and 37 °C [30]. To form additional networks within the collagen structure, additional macromolecules must be mixed with collagen before the initiation of fibrillogenesis or interpenetrate its structure after its initial curing. We combined both approaches. To form fibrin structures, fibrinogen was mixed with the collagen and HA precursor solution and polymerized in the presence of thrombin. HA is physically entrapped within the Col I and fibrin networks, while PEGDA chains of varying lengths are polymerized by exposure to UV radiation. Our team has successfully characterized mIPN containing HA, Col I, and PEGDA over a broad range of concentrations and a discrete number of MWs [22,23]. The incorporation of fibrin into our existing platform was explored, starting with fabrication conditions previously reported in the literature. Fibrin has been incorporated in hydrogels used for tissue-engineering applications at concentrations as high as 50 mg/mL [31]. However, for modeling the abnormal and elevated presence of fibrin in CNS tissue, the fibrin concentration levels do not need to approach this upper bound previously reported in other tissue-engineering applications. To select a relevant range of fibrin levels in our model, we considered the normal physiological levels of fibrinogen in human blood and the increased values reported in TBI patients [32,33]. We also considered histological analyses of total protein and relative fibrin levels in cortex tissue [15,34,35].

#### 2.1.1. Tuning Thrombin-Fibrinogen Ratio

To form a fibrin structure, fibrinogen molecules must be combined with thrombin. The ratio of thrombin and fibrinogen determines the gelation time and the mechanical properties of the resulting hydrogels [36]. One of our preliminary objectives was to tune the thrombin-fibrinogen ratio so that the gelation could take place within 30 minutes. Successful gel formation was evaluated by observing the increase in the opacity of the precursor solution and the absence of flow of the precursor under a 45° inclination angle. Under normal physiological conditions in human blood, fibrinogen concentrations range from approximately 1.5 to 4.0 g/L [32]. However, in patients 2 days after injury, these levels exceed the normal range and remain elevated for as long as 14 days [33]. It has also been reported that in human brain cortex tissue, the total protein concentration ranges from 42 to 46 mg/g of tissue [34]. In addition, in an AD model, fibrin levels were found to be between 1 and 5 µg fibrin/mg protein [15]. Fibrin levels may increase 20-fold to 100-fold in diseased tissue relative to healthy tissue [35]. Therefore, in this work, we decided to explore the presence of fibrin in mIPNs at levels of 0.0, 2.0, 4.0, 6.0, and 8.0 mg/mL. The selected concentrations will cover the potential range of levels of fibrin present in normal and diseased tissue. To tune the thrombin-fibrinogen ratio, we selected the 6.0 mg/mL formulation. The use of one of the high fibrinogen concentration precursor solutions facilitated the qualitative assessment of the gel formation. Table 1 summarizes the results of the tuning of the thrombin-fibrinogen ratio.

As expected, the increase in the thrombin-fibrinogen ratio resulted in faster curing time, even at room temperature. The use of 0.1 U/mg fibrinogen did not yield a gel structure after 30 min at 37 °C. However, a longer curing time could allow the precursor solution to transition into a gel phase. For our fabrication approach, 0.2 U/mg of fibrinogen seems to be the most appropriate level and compatible condition with the collagen crosslinking strategy previously used by our research team [22]. Using this thrombin-fibrinogen ratio allowed the curing of the fibrin network at a rate that permitted the resuspension of living cells within the collagen-based mIPN solution. The use of a 0.2 U/mg fibrinogen ratio was also within the range of values previously reported in the literature [36,37].

#### 2.1.2. Incorporating Varying Fibrin Levels on Hydrogel Mechanical Performance

**Figure 1:**
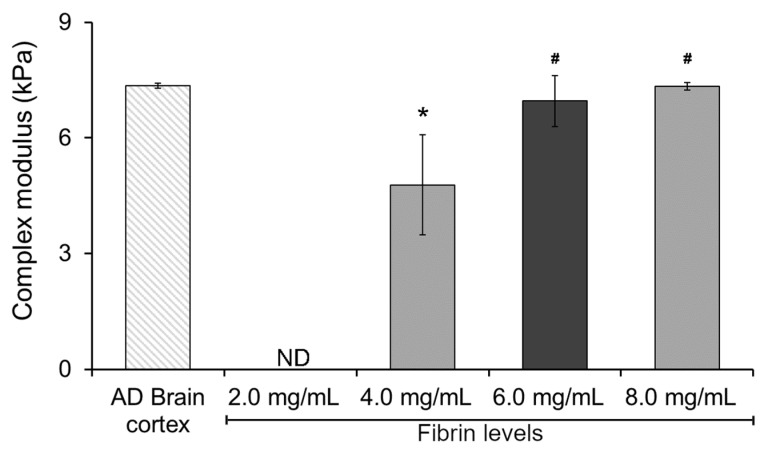
The effect of increasing fibrin concentration on the complex modulus of Col I-fibrin IPNs; Col I levels were maintained consistently at 3.0 mg/mL across all formulations. *, significantly different from AD brain cortex, *p* < 0.05; #, significantly different from mIPN 1, *p* < 0.05, n = 4. ND, below the detection limit of the testing method.

To gain insight and understand the potential effect of fibrin levels on the mechanical performance of our hydrogel system, we started by characterizing the complex modulus of a double-network Col I-fibrin hydrogel using dynamic mechanical analysis (DMA). This initial assessment allowed us to narrow down the potential range of fibrin levels within our mIPNs. As expected, increasing fibrin levels resulted in hydrogels exhibiting increasing values of complex modulus (Figure 1). This finding aligns with previous literature on fibrin hydrogels, indicating that heightened fibrin concentration can enhance crosslinking and create a more compact 3D network, resulting in increased stiffness and elastic energy storage and, therefore, raising the complex modulus [38,39]. The complex modulus of the 2.0 mg/mL Col I-fibrin hydrogel was below the detection level of our testing methods. Moreover, relative to the AD brain cortex control group, the complex modulus of the 4.0 mg/mL Col I-fibrin IPN was significantly lower (1.54-fold, *p* = 0.011). Differences among the 6.0 and 8.0 mg/mL fibrin formulations and the AD human brain cortex group were not statistically different (*p* ≥ 0.931). Finally, relative to the 4.0 mg/mL group, the complex modulus of the 6.0 and 8.0 mg/mL groups increased significantly to approximately 1.46- and 1.54-fold (*p* ≤ 0.028).

These data demonstrated that the resulting double-network Col I-fibrin hydrogel could achieve similar mechanical performance as those in diseased brain cortex tissue and that hydrogels with fibrin levels above 6.0 mg/mL already achieved a complex modulus similar to that of the AD brain cortex sample (7.35 ± 0.06 kPa), therefore limiting the introduction of additional components into the mIPNs. Consequently, we selected a fibrin concentration of 6.0 mg/mL as the maximum value for the screening of a library of mIPN formulations.

#### 2.1.3. Tailoring the Mechanical Properties of mIPNs

The viscoelastic properties of 3D cell culture substrates play a significant role in regulating cellular functions such as proliferation, migration, and differentiation [40]. Our main aim was to develop a series of scaffolds that could closely resemble the mechanical properties of human AD brain cortex tissue while varying the levels of fibrin and hyaluronic acid in the mIPN matrix. By achieving this goal, we could potentially use this platform to elucidate the effects of a reduction in HA content and an increase in the presence of fibrin independently from matrix stiffness on the cellular response of CNS cells. A decrease in HA and the abnormal presence of fibrin in CNS tissue have been previously identified as key neuroinflammatory triggers [41,42]. The potential establishment and validation of the proposed in vitro platform will contribute to our understanding of cellular mechanisms that regulate inflammatory signaling in CNS tissue. Previous studies have demonstrated that the mechanical properties of collagen, HA, and PEGDA mIPNs can be adjusted by modifying their chemical compositions and that by adjusting PEGDA molecular weight and concentration the mechanical properties of the scaffold can be adjusted to closely match the mechanical properties of cortex tissue [18,43]. The current study uses a similar strategy to increase the complexity and chemical landscape of the platform by introducing fibrin. Herein, we fabricated and characterized the mechanical performance of 12 different hydrogels. Initial assessment of the fabrication conditions of hydrogels containing constant levels of Col I (3.0 mg/mL) and varying levels of fibrin (2.0, 4.0, 6.0, 8.0 mg/mL) indicates that the results were in close proximity to the modulus target value. Our next step was focused on the introduction of PEGDA into the mIPNs to promote the physical stability of the scaffolds and to finely tune their complex modulus. Different PEGDA molecular weights (3.4 kDa, 6.0 kDa, and 10.0 kDa) and varying concentrations ranging from 5.26% *w*/*w* to 6.25% *w*/*w* were explored. Additionally, HA, which is abundantly found in CNS tissue, was incorporated at 2.0 mg/mL into the mIPNs 9–12, as detailed in Table 2. It is worth noting that the selected levels of PEGDA and HA have been previously employed in our studies [22,23] and were adjusted based on the expertise of our research group to achieve the target value of complex modulus.

As expected, the mechanical data results in Table 2 showed that in the absence of other compositional changes, the average complex modulus of the scaffolds overall appears to increase with rising levels of fibrin (mIPN 1–4, mIPN 6–8 and mIPN 10–12). The increase in modulus can be attributed to the increase in the packing of the fibrin network as its concentration is elevated across the different mIPN formulations. However, significant differences were only observed in scaffolds that did not contain HA and PEGDA (*p* ≤ 0.005).

In the absence of hyaluronic acid (HA), 10.0 kDa PEGDA was employed at 6.25% *w*/*w* to achieve the target complex modulus. In the mIPN 6–8 series, the PEGDA molecular weight was reduced from 10.0 kDa to 6.0 kDa to facilitate the diffusion of PEGDA molecules into the scaffold. The concentration was reduced from 6.25% w/w to 5.26% *w*/*w* to avoid an overshoot in the complex modulus. Despite using PEGDA with the same molecular weight and concentration in these series, the complex modulus remained close to that of the AD brain cortex when fibrin concentration increased. We speculated that this could be attributed to reduced scaffold permeability, influenced by the combined effects of increased scaffold hydrophobicity and elevated fibrin fibril density as fibrin levels (moderately hydrophobic) rise [42,43].

In the formulations containing HA (mIPN 9–12), 3.4 kDa and 6.0 kDa PEGDA molecular weights were employed to achieve the target complex modulus. The presence of HA as an additional component in mIPN 9–12 also seems to reduce the permeability of the scaffolds. This is evident in the demand for a lower PEGDA molecular weight in the 0.0 mg/mL fibrin condition relative to mIPN 5 to maintain a complex modulus of around 7.35 kPa. These results suggest that the incorporation of HA may reduce the permeability of scaffolds as the overall multi-network structure becomes densely packed. Importantly, statistical analysis indicates no significant differences between the control group (AD brain cortex tissue) and mIPN 3–12 (*p* ≥ 0.913).

### 2.2. Microarchitecture of mIPNs

**Figure 2:**
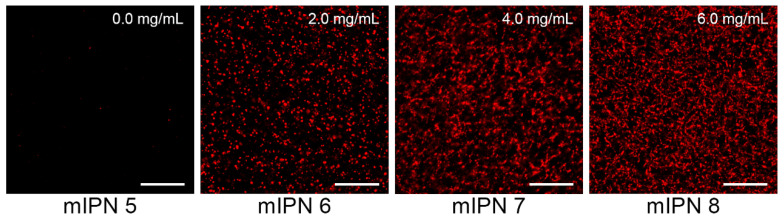
Representative confocal images of fibrin structure of mIPNs 5–8 at different fibrin concentrations (0.0–6.0 mg/mL); scale bar = 50 µm.

The microarchitecture of mIPNs was characterized using standard microscopy techniques. We performed immunostaining followed by confocal laser scanning microscopy (CLSM) and scanning electron microscopy (SEM) to identify, confirm, and visualize the distribution of Col I, fibrin, and HA within the mIPNs. The confocal images shown in Figure 2 confirmed the presence of fibrin within our Col I-fibrin-PEGDA mIPN. Images also revealed an increase in the packing density of fibrin, as the concentration of fibrin increased from 0.0 to 6.0 mg/mL in mIPNs 5–8. This observation aligns with previous reports [37,39] and is directly linked to the apparent increase in mechanical properties discussed earlier in relation to mIPNs 5–8.

**Figure 3:**
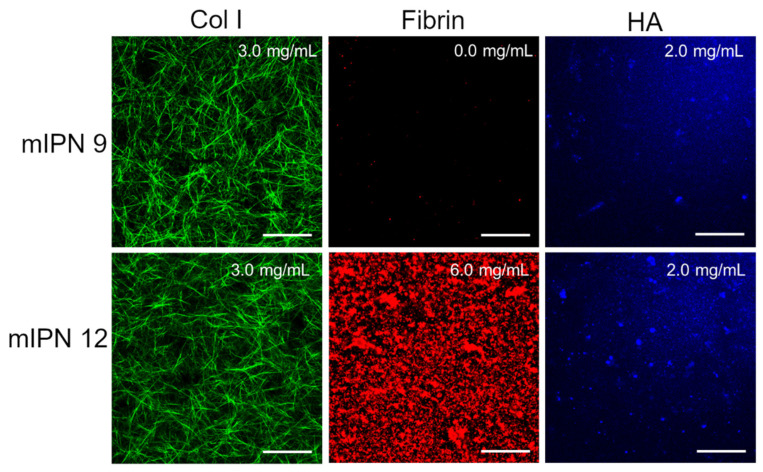
Representative confocal images of mIPN containing Col I (3.0 mg/mL), HA (2.0 mg/mL), and fibrin (0.0 mg/mL and 6.0 mg/mL); scale bar = 50 µm.

Furthermore, by characterizing mIPN 9 and mIPN 12 using immunostaining, we aimed to demonstrate that the introduction of the highest levels of fibrin proposed in this exploratory study did not significantly change the collagen structure and HA distribution (Figure 3). Relative to mIPN 8 (Figure 2), which does not contain HA, fibrin structures are more densely packed when HA has been incorporated within the scaffold. We speculate that the strong hydrophilic nature of HA induces fibrin accumulation. Fibrin is hydrophobic upon its conversion by thrombin [44].

**Figure 4:**
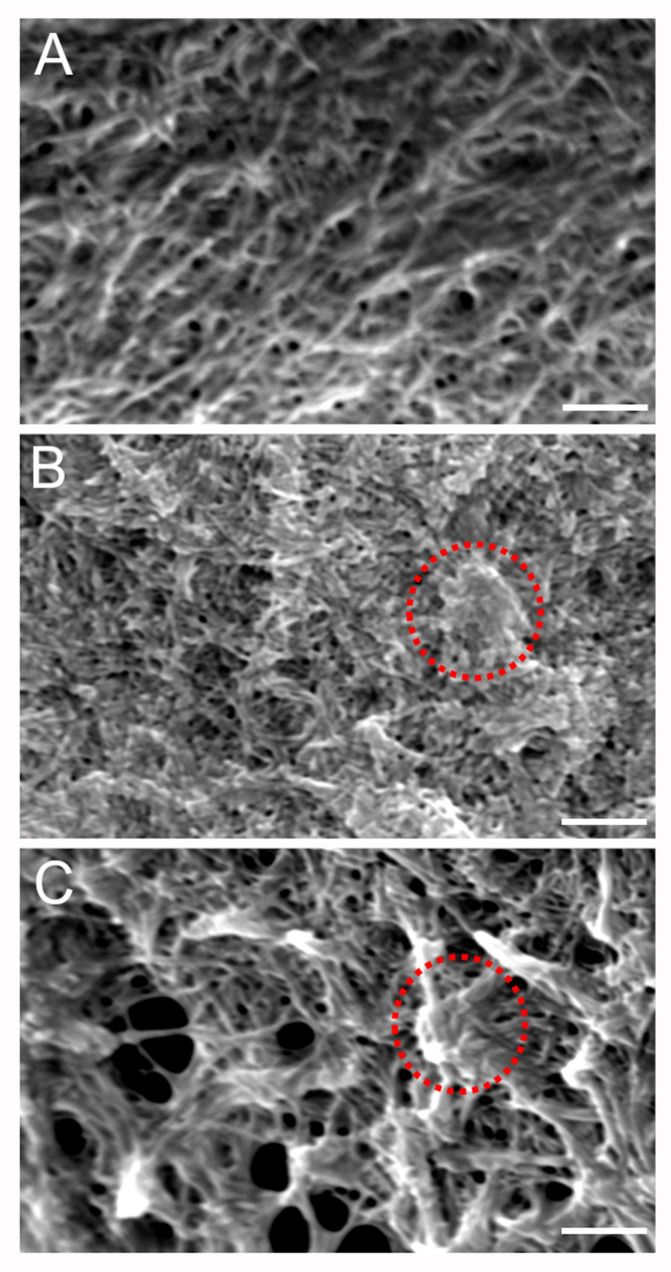
Representative SEM micrographs of scaffold microstructure. (**A**) Col I-HA (2.0 mg/mL)-PEGDA. (**B**) Col I-fibrin (6.0 mg/mL)-PEGDA (**C**) Col I-HA (2.0 mg/mL)-fibrin (6.0 mg/mL)-PEGDA; Scale bar = 2 µm; 10 kV and 7000× magnification. The red circles highlight denser areas or nodes associated with the introduction of fibrin.

To gain further insight into the microstructural organization of the different components within the mIPN, SEM analysis was performed on three different formulations. Figure 4A shows the microstructure of mIPN 9, which contains Col I, HA, and PEGDA. The micrograph revealed collagen fiber embedded within the HA, closely resembling previous results from Van Drunen et al. [22]. The microscopic landscape of mIPN 5, as shown in Figure 4B, exhibits a fibrous appearance attributed to the presence of collagen fibers. Notably, the introduction of fibrin is evident through denser areas or nodes within the structures, as highlighted by circles in Figure 4. These thick fibrin fibers are identified as aggregates of protofibrils, which are difficult to distinguish from one another and have been reported in prior studies to be the characteristic appearance of fibrin hydrogels at concentrations such as 6 mg/mL [45]. Finally, in mIPN 12, a distinct combination of both microarchitectures is evident due to the addition of fibrin and HA to the hydrogel. A microstructure characterized by fibers, porous features, and fibrin nodes is observed. These findings suggest that the fibrin is well incorporated within the mIPN structure and the microstructure, confirming the observations highlighted using CLSM.

### 2.3. Cell Viability

**Figure 5:**
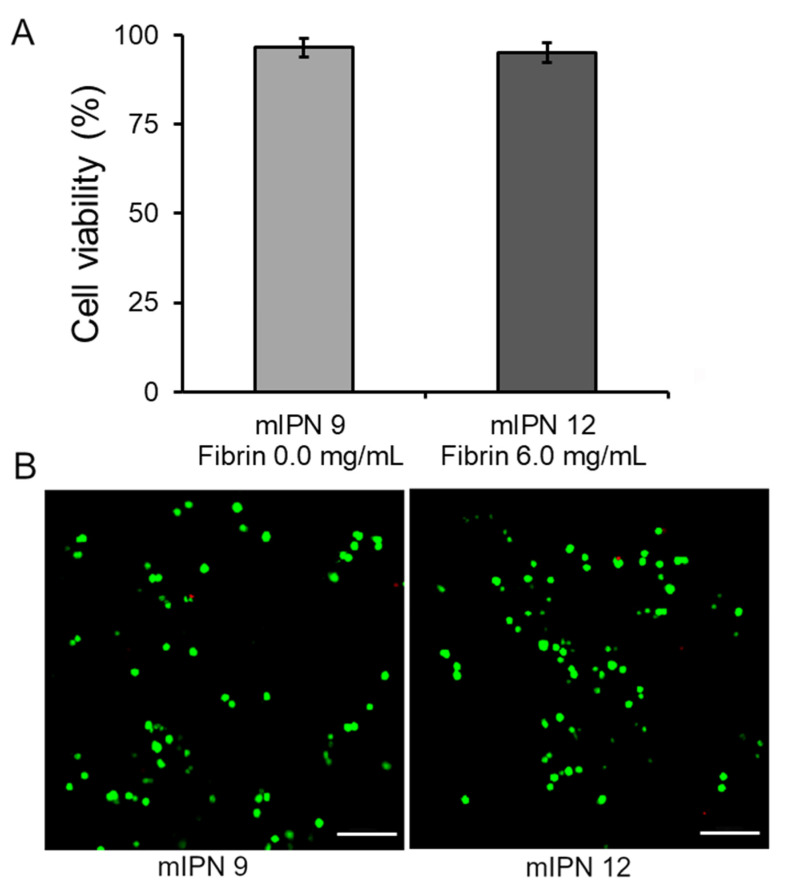
Cell viability of human astrocytes after 1 day of culture in mIPN 9 and mIPN 12. (**A**) Quantitative results using ImageJ software version 1.54f. (**B**) Representative laser scanning confocal microscopy images of Live/Dead staining, n = 7; scale bar = 100 µm.

Our final goal was to assess that our fabrication procedure yielded cytocompatible scaffolds capable of supporting the 3D in vitro culture of human astrocyte cells, enabling future studies on cellular responses. Toward this end, the live/dead viability assay was performed 24 h post-cell encapsulation in the mIPN 9 and mIPN 12. The assay determined the ratio of live cells stained with green fluorescence to the total number of cells. The results indicated that the cell viability was 96.5 ± 2.6% for mIPN 9 and 95.2 ± 2.8% for mIPN 12 (Figure 5A), with no statistically significant difference in mean values. The viability of astrocytes was observed to be consistent even after 24 h of fabrication, regardless of the fibrin content in the hydrogels. These findings suggest that mIPNs and the fabrication method do not impair cell viability and offer support for this type of CNS cells. Notably, these results agree with previous studies of encapsulated CNS cells in hydrogels comprising similar components, which also reported conserved cell viability [46]. These results also agree with previously reported results from our laboratory [22].

### 2.4. Qualitative Assessment of Cell Morphology

**Figure 6:**
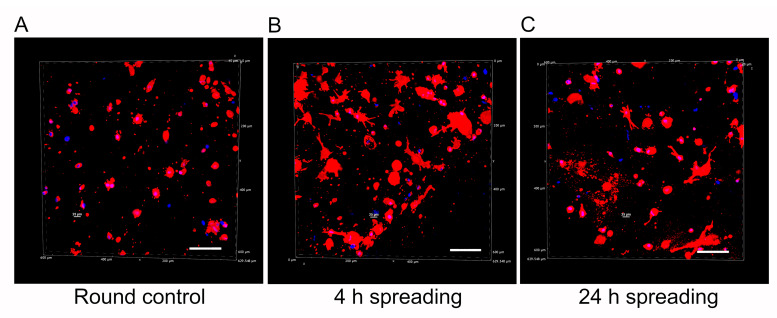
Representative 3D reconstructions of DAPI/phalloidin-stained human cortex astrocytes in mIPN 12 as a function of the spreading time before PEGDA infiltration. (**A**) Round control. (**B**) 4 h spreading time. (**C**) 24 h spreading time. The cell cytoskeleton is stained in red with rhodamine-phalloidin, and the cell nuclei are stained in blue with DAPI; scale bar = 100 µm.

An inherent challenge in advancing biomaterials for astrocyte research lies in achieving a physiological cell morphology during culture [47]. In alignment with this challenge and our final goal, we performed a qualitative analysis of human astrocyte morphology within the mIPN 12 scaffold, characterized by its four components and the highest fibrin concentration. The aim of this analysis was to elucidate the capacity of scaffolds with multiple components to facilitate effective cell spreading. To highlight changes in cell morphology over time, human astrocytes were allowed to spread for 30 min, 4 h, and 24 h before PEGDA infiltration and subsequent photopolymerization. Confocal microscopy images of human astrocytes encapsulated in mIPN 12 are shown in Figure 6. Cell spreading visibly increased with time, using the 30-minute spreading period as the control group. Astrocytes in the control group exhibited round morphologies, while those allowed to spread for 4 h and 24 h displayed a more elongated morphology. Some exhibited a characteristic star-shaped appearance, confirming the ability of mIPNs to support cell spreading. In contrast to our prior study using Col I, HA, and PEGDA [23], which determined that a 4 h spreading time was the maximum duration without significant bulk matrix contraction, our current findings underline the dependency of spreading time on matrix composition. For our mIPN 12, an elongated morphology was initially observed 4 h after the encapsulation of the cells, and no visible contraction of the scaffold was evident even at 24 h. In addition, a previous study using similar hydrogel systems containing Col I, HA, and PEGDA confirmed a spread morphology for human astrocytes after 7 and 14 days in culture [21].

In terms of scaffold stability, we hypothesize that our quad-component system will exhibit similar physical stability as previously reported in the literature. For instance, Munoz-Pinto et al., in their assessment of resistance to cell-mediated compaction, monitored changes in collagen-PEGDA IPN dimensions over 14 days of culture of elongated human mesenchymal stem cells (hMSCs). Notably, no alteration in IPN thickness was observed, and the cell shapes were consistently maintained through the culture period. These results were consistent with the resistance to cell-mediated compaction characteristic of pure PEGDA hydrogels. In contrast, collagen-based scaffolds supporting the 3D growth of hMSCs after 14 days of culture exhibited a significant reduction in the construct diameter of approximately 60% relative to day 0 [20]. These findings emphasize the advantageous effects of incorporating PEGDA into hydrogel scaffolds, especially those comprising natural components. The addition of PEGDA not only enhances the mechanical properties of the scaffold but also can improve its physical stability over an extended culture period [28].

## 3. Conclusions

In this work, we successfully demonstrated that the proposed materials and a network-by-network fabrication strategy can be used to create complex composite materials in which Col I, HA, fibrin, and PEGDA networks are interpenetrated in a single hydrogel-based structure. We also showed that this hydrogel platform allowed for the modulation of the complex modulus and that the complex modulus can be tuned to match the mechanical performance of AD human brain cortex tissue. The microstructural characterization confirmed that increasing levels of fibrin can be incorporated within the scaffolds and that the individual components for the mIPNs were successfully intertwined within one single macroscopic structure. Finally, we observed that the proposed network-by-network fabrication approach yields a biomaterial with at least 95% cell viability using astrocytes of human origin. Furthermore, it is noteworthy that the scaffolds, even with multiple components, also enable the spreading of human astrocytes. Future studies will be focused on the use of the proposed platform to study the effect of abnormal fibrin levels in CNS tissue on the biological response of human astrocytes and other human CNS cells.

## 4. Materials and Methods

### 4.1. mIPNs Fabrication

A library of mIPN formulations was characterized to identify different compositions in which the levels of hyaluronic acid and fibrin could be modulated while displaying a complex modulus similar to that exhibited in human diseased brain cortex tissue. The composition of several mIPNs is summarized in Table 2.

The mIPNs were fabricated using a modified protocol for the network-by-network fabrication approach previously described by Van Drunen et al. [22]. In brief, three precursor solutions were prepared:

- Solution A: collagen type I at a final concentration of 6.0 mg/mL in 1× phosphate-buffered saline (PBS) was obtained after the neutralization of collagen type I from rat tail at a concentration of 9.5 mg/mL (Corning, Bedford, MA, USA), using 1N NaOH (Amresco, Solon, OH, USA) and 10× PBS (Lonza, Walkersville, MD, USA). This solution was stored on ice until use.
- Solution B: fibrinogen (FG, Alfa Aesar, Ward Hill, MA, USA) at 4× the target fibrin levels in 1× PBS. This solution was prepared fresh at room temperature until the complete dissolution of FG, then sterile-filtered and stored on ice until use.
- Solution C: high molecular weight HA (HMW-HA; 1010 kDa–1800 kDa, Lifecore Biomedical, Chaska, MN, USA) at 4× the target concentration and appropriate thrombin levels to achieve 0.2 U of thrombin/mg of FG in 1× PBS. HMW-HA was dissolved at 4 °C for 12 h. The solution was sterile-filtered, and thrombin was added. Once thrombin was added, the solution was stored on ice until use.

**Figure 7:**
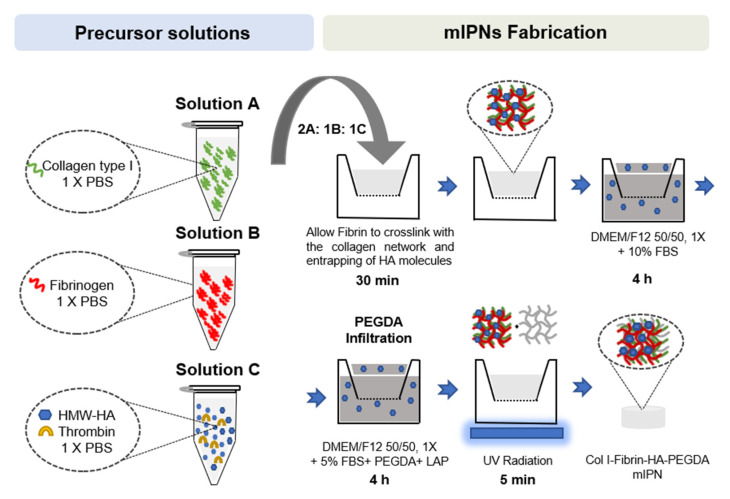
Schematic representation of mIPNs fabrication process.

Once all precursor solutions were prepared, solutions A, B, and C were carefully mixed on ice at a 2:1:1 ratio, respectively. Once all components were well mixed, 75 µL of mIPN precursor solution was pipetted into 24-well culture inserts (8 μm pore size, Greiner Bio-One, Monroe, NC, USA) and allowed to physically crosslink at 37 ℃ and 5% CO_2_ in the cell culture incubator for 30 min. Following the crosslinking of the Col I and fibrin and the entrapping of HA molecules, Dulbecco’s Modified of Eagle’s Medium/ F-12 without phenol red (DMEM/F12 50/50, 1×, Corning, Manassas, VA, USA) supplemented with 10% fetal bovine serum (FBS, Atlas Biological, Fort Collins, CO, USA), and 1% penicillin-streptomycin (PS, Gibco, Grand Island, NY, USA) was added above and below the inserts. To ensure that the physically intertwined HMW-HA molecules remained contained in the scaffolds, the cell culture media was supplemented with HMW-HA, matching the levels of HA in the target mIPN. The constructs were then placed in the cell culture incubator for 4 h. Following the incubation period, the culture media was replaced with an infiltration solution composed of DMEM/F12 50:50, 1X without phenol red, supplemented with 5% FBS and 1% PS, PEGDA of varying molecular weight and concentration, and 1 mM of lithium phenyl-2,4,6-trimethylbenzoylphosphinate (LAP) as photoinitiator and HMW-HA when appropriate. After a 4 h incubation period, the PEGDA infiltrating solution was carefully removed, and the constructs were exposed to 365 nm UV radiation for 5 min using a TL-365R Spectroline UV transilluminator (Spectronics Corporation, Westbury, NY, USA). PEGDA and LAP were synthesized as we have described in previous works [48,49]. A schematic representation of the mIPN fabrication process is shown in Figure 7.

### 4.2. Cell Culture and Encapsulation in mIPNs

Human hippocampus astrocytes (hA, Celprogen, Torrance, CA, USA) were cultured and expanded in DMEM/F12 (50:50) medium supplemented with 10% FBS; (Atlas Biological, Fort Collins, CO, USA) 1% Glutamax (Gibco, Grand Island, NY, USA) and 1% penicillin/streptomycin (Gibco, Grand Island, NY, USA). The medium was replaced every 2 days, and the cell culture was expanded at 37 °C and 5% CO_2_ until use (passage 5–6). Human brain cortex astrocytes (StemBioSys, San Antonio, TX, USA) were cultured and expanded until use (passage 5–6) on poly-L-lysine coated tissue culture flasks, astrocyte medium (ScienCell Research Laboratories, Carlsbad, CA, USA) at 37 °C and 5% CO_2_. The cells were encapsulated under sterile conditions by resuspending approximately 1.0 × 10^6^ cells/mL in the ice-cold Col I-fibrin-HA solution to continue with scaffold fabrication, as described in Section 4.1.

### 4.3. Characterization of mIPN Mechanical Properties

The mechanical characterization of the scaffolds was performed by dynamic mechanical analysis (DMA). After 24 h, the mechanical properties of the mIPNs were characterized at room temperature using a TA Instruments Electroforce 3100 mechanical tester (TA Instruments, Eden Prairie, MN, USA). Each mIPN formulation was fabricated in the absence of cells according to the procedure described above and tested using five independent specimens of approximately 8.0 mm in diameter and 1.5 mm in height. The specimens were removed from their inserts with a small plastic spatula, measured, and placed gently on the instrument preloaded with a 2 g force and exposed to oscillations of 100 μm in amplitude at 1 Hz of frequency. The complex modulus (E*) was calculated as follows:

(1) $$E^{*}=\sqrt{E^{\prime 2}+E^{\prime \prime 2}}$$

where *E*′ and *E*″ are the modulus of storage and loss, respectively. Human brain cortex samples from Alzheimer’s disease (AD) patients were used as the control group. The control samples were facilitated by the Department of Pathology at Case Western Reserve University. Human brain tissue was obtained with informed written consent of relatives from autopsy AD patients for neuropathology examination. All protocols used in this study were approved by the Bioethics Committee (Department of Pathology, Case Western Reserve University). All methods were carried out in accordance with the approved guidelines and regulations. Cortex samples were cut using a sterile 8 mm biopsy punch (Integra Miltex, York, PA, USA) to achieve a similar sample geometry as in the mIPN samples.

### 4.4. Microarchitectural Characterization

Microarchitectural characterization of the mIPNs was performed using confocal laser scanning microscopy (CLSM) and scanning electron microscopy (SEM), as outlined below. Images from CLSM allow the confirmation of the presence and distribution of the main natural macromolecules within the mIPN. For confocal images, approximately 1 mm thick transverse sections from each mIPN hydrogel were manually cut and fixed with 10% formalin (VWR, Radnor, PA, USA) for 1 h at room temperature. To visualize the collagen, HMW-HA, and fibrin structures, we stained the hydrogels with ECM-binding agents. The hydrogel sections were washed three times with PBS and blocked with 3% bovine serum albumin (BSA, Fair Lawn, NJ, USA) in PBST (PBS plus 0.05% tween 20) for 1 h. Samples were then exposed for 12 h at 4 °C to primary antibodies or the HA binding protein diluted in the staining buffer (PBST containing 3% BSA). For collagen I, we used Col 1A mouse monoclonal IgG, 1:200 dilution (Santa Cruz Biotechnology, Dallas, TX, USA), and for fibrin, we used rabbit anti-human fibrinogen,1:200 dilution (Agilent Technologies, Santa Clara, CA, USA). The presence of HMW-HA was characterized by exposing the samples to biotinylated hyaluronic acid binding protein (MilliporeSigma, Burlington, MA, USA, 1:100). The hydrogels were subsequently rinsed three times with PBS and secondary antibodies (Anti-Mouse Alexa Fluor^®^ 488 Conjugate, 1:50, Invitrogen, Rockford, IL, USA; Rhodamine Red anti-rabbit, 1:50 Life Technologies, Eugene, OR, USA; and Streptavidin Alexa Fluor 405 conjugate, 1:50, Invitrogen) diluted in the staining buffer were applied for 12 h at 4 °C. Following incubation, the slices were washed three times with PBS to remove unbonded secondary antibodies or streptavidin. Confocal microscopy was performed using a Nikon A1 confocal microscope system with a 20× objective (Nikon, Melville, NY, USA). Images were captured by selecting three random regions per sample and seven consecutive shots in the z direction per region.

SEM micrographs of the scaffold microstructures were obtained using a JEOL LA scanning electron microscope (JEOL, Peabody, MA, USA). The effects of adding fibrin, incorporating hyaluronic acid, and combining fibrin and HA were investigated on mIPN 8, mIPN 9, and mIPN 12. To prepare the samples for imaging, they were fixed with formalin and washed three times with water. The samples were subsequently frozen in N_2_(L) and lyophilized for 24 h. Prior to imaging, dried hydrogels were mounted on SEM sample stages using carbon tape and sputter-coated with gold to a thickness of approximately 6 nm using a Cressington 108 auto sputter coater (Cressington Scientific Instruments, Ted Pella Inc., Redding, CA, USA). SEM micrographs (n = 5) were taken from the cross-sectional area of each specimen at 10 kV and 7000× magnification.

### 4.5. Cell Viability Assessment

The live/dead cell viability assay (Invitrogen, Life Technologies, Eugene, OR, USA) was conducted to evaluate the initial effects of the mIPN fabrication process on cell viability. The assay was performed 24 h after the cell encapsulation in the networks containing Col I, HMW-HA, and PEGDA, both without fibrin and with the highest fibrin concentration, mIPN 9 and mIPN 12, respectively. Ethidium homodimer-1 (Ethd-1) and calcein AM were employed in accordance with the manufacturer’s protocol. At least three independent specimens and seven random images were recorded for each network using a laser Nikon A1 confocal microscope system (Nikon, Melville, NY, USA). The images were processed using ImageJ software (National Institutes of Health, USA, version 1.54f), and the live and dead cells were quantified. The viability was reported as the ratio of live cells stained with green fluorescence to the total number of cells.

### 4.6. Cell Morphology Characterization

Cell morphology was characterized using confocal laser scanning microscopy. In brief, cells were allowed to spread in mIPN 12 for 30 min, 4 h, and 24 h before initiating the diffusion of PEGDA. Once the gels were obtained, approximately 1 mm transverse sections from each time point were manually cut and fixed with formalin. The sample slices were washed three times with PBS and stained using rhodamine-phalloidin (Life Technologies, Eugene, OR, USA) at a 1:100 dilution and DAPI dilactate, 4′,6-diamidino-2-phenylindole dilactate, (Life Technologies, Eugene, OR, USA) at a 1:300 dilution. Following overnight incubation at 4 °C, the slices were washed three times with PBS and placed in a 35 mm glass bottom dish surrounded by a small amount of PBS to prevent dehydration during the imaging. Image acquisition was performed using a Nikon A1 confocal microscope system with a 20× objective (Nikon, Melville, NY, USA). Three random regions were selected for imaging per sample, with six consecutive shots in the z direction per region.

### 4.7. Statistical Analysis

Data results are reported as mean plus or minus the standard deviation. Statistical analysis was performed using an ANOVA followed by Tukey’s post-hoc test (IBM SPSS Statistics software, version: 29.0.1.0). Statistical significance was set at *p* < 0.05.

## Figures and Tables

**Table 1:** The effect of the thrombin-fibrinogen ratio on the formation of a 6.0 mg/mL fibrin hydrogel in 1X PBS.

Fibrinogen [mg/mL]	Thrombin [U/mg fibrinogen]	T [°C]	Curing Time [min]	Hydrogel Formation
6.0	1.0	25	0.5 ^1^	Successful
6.0	0.4	25	1.0 ^1^	Successful
6.0	0.2	37	30.0	Successful
6.0	0.1	37	30.0	Unsuccessful

^1^ The crosslinking of the fibrin network took place before transferring the precursor solution to the cell culture incubator.

**Table 2:** Composition of mIPN containing Col I at 3.0 mg/mL.

Formulation	HA	Fibrin	PEGDA		Complex Modulus
	[mg/mL]	[mg/mL]	[kDa]	[%*w*/*w*]	[kPa]
AD Brain cortex	-	-	-	-	7.35 ± 0.06
mIPN 1	0.0	2.0	0.0	0.00	ND
mIPN 2	0.0	4.0	0.0	0.00	4.78 ± 1.30 *
mIPN 3	0.0	6.0	0.0	0.00	6.96 ± 0.66 ^#^
mIPN 4	0.0	8.0	0.0	0.00	7.34 ± 0.10 ^#^
mIPN 5	0.0	0.0	10.0	6.25	7.26 ± 1.42
mIPN 6	0.0	2.0	6.0	5.26	7.28 ± 1.44
mIPN 7	0.0	4.0	6.0	5.26	7.73 ± 0.38
mIPN 8	0.0	6.0	6.0	5.26	8.47 ± 1.59
mIPN 9	2.0	0.0	3.4	5.75	7.23 ± 1.42
mIPN 10	2.0	2.0	6.0	5.75	6.58 ± 0.53
mIPN 11	2.0	4.0	6.0	5.75	6.34 ± 2.02
mIPN 12	2.0	6.0	6.0	5.75	7.24 ± 0.99

Data are reported as averages plus or minus the standard deviation, n = 5. *, significantly different from brain cortex; ^#^, significantly different from mIPN 1, *p* < 0.05.

## Data Availability

The original contributions presented in the study are included in the article, further inquiries can be directed to the corresponding authors.
